# Differentially expressed miRNAs in triple negative breast cancer between African-American and non-Hispanic white women

**DOI:** 10.18632/oncotarget.13024

**Published:** 2016-11-02

**Authors:** Bruna Sugita, Mandeep Gill, Akanskha Mahajan, Anju Duttargi, Saurabh Kirolikar, Rodrigo Almeida, Kenny Regis, Olusayo L. Oluwasanmi, Fabio Marchi, Catalin Marian, Kepher Makambi, Bhaskar Kallakury, Laura Sheahan, Iglenir J. Cavalli, Enilze M. Ribeiro, Subha Madhavan, Simina Boca, Yuriy Gusev, Luciane R. Cavalli

**Affiliations:** ^1^ Department of Genetics, Federal University of Paraná, Curitiba, PR, Brazil; ^2^ Department of Oncology, Lombardi Comprehensive Cancer Center, Georgetown University Medical Center, Washington, DC, USA; ^3^ International Research Center-CIPE, A. C. Camargo Cancer Center, São Paulo, SP, Brazil; ^4^ The Ohio State University Comprehensive Cancer Center, Division of Cancer Prevention and Control, College of Medicine, The Ohio State University, Columbus, Ohio; ^5^ Departments of Biostatistics, Bioinformatics, and Biomathematics, Georgetown University, Washington, DC USA; ^6^ Department of Pathology, Georgetown University Medical Center, Washington, DC, USA; ^7^ Innovation Center for Biomedical Informatics, Lombardi Comprehensive Cancer Center, Georgetown University Medical Center, Washington, DC, USA; ^8^ The University of Medicine and Pharmacy Timisoara, Timisoara, Romania

**Keywords:** microRNA, triple negative breast cancer, African American, non-Hispanic white, copy number

## Abstract

Triple Negative Breast Cancer (TNBC), a clinically aggressive subtype of breast cancer, disproportionately affects African American (AA) women when compared to non-Hispanic Whites (NHW). MiRNAs(miRNAs) play a critical role in these tumors, through the regulation of cancer driver genes. In this study, our goal was to characterize and compare the patterns of miRNA expression in TNBC of AA (*n* = 27) and NHW women (*n* = 30). A total of 256 miRNAs were differentially expressed between these groups, and distinct from the ones observed in their respective non-TNBC subtypes. Fifty-five of these miRNAs were mapped in cytobands carrying copy number alterations (CNAs); 26 of them presented expression levels concordant with the observed CNAs. Receiving operating characteristic (ROC) analysis showed a good power (AUC ≥ 0.80; 95% CI) for over 65% of the individual miRNAs and a high combined power with superior sensitivity and specificity (AUC = 0.88 (0.78−0.99); 95% CI) of the 26 miRNA panel in discriminating TNBC between these populations. Subsequent miRNA target analysis revealed their involvement in the interconnected PI3K/AKT, MAPK and insulin signaling pathways. Additionally, three miRNAs of this panel were associated with early age at diagnosis. Altogether, these findings indicated that there are different patterns of miRNA expression between TNBC of AA and NHW women and that their mapping in genomic regions with high levels of CNAs is not merely physical, but biologically relevant to the TNBC phenotype. Once validated in distinct cohorts of AA women, this panel can potentially represent their intrinsic TNBC genome signature.

## INTRODUCTION

Triple negative breast cancer (TNBC) is a clinically aggressive subtype of breast cancer that confers a high risk of metastasis development, usually shortly after the initial diagnosis [1]. Despite numerous advances identifying and testing potential biomarkers and their corresponding therapeutic compounds in TNBC clinical trials, an effective and approved targeted therapy for these tumors is not yet available [2, 3].

Variation among different racial and ethnic groups in the incidence of breast cancer molecular subtypes and clinical outcomes is well documented [4, 5]. The basal-like tumors, in particular TNBC, are present at higher frequencies in African American (AA) women when compared to non-Hispanic Whites (NHW), Hispanics or Asian women [5]. AA women with TNBC disease are usually diagnosed at an earlier age and more advanced stage, and likely to develop early metastasis compared to NHW women [5]. An increased number of studies have characterized the differences in tumor biology between AA and NHW patients and have shown that breast tumors from AA patients present increased cell proliferation, elevated expression of angiogenesis markers and higher migration and invasive properties [6–8]. These findings suggest that although socio-economic and cultural factors play a role, biological factors can also be the major drivers of these disparities [8, 9].

MicroRNAs (miRNAs) are a class of non-coding endogenous RNA molecules that have been identified to play a role in breast cancer, through the regulation of cellular processes associated with aggressive tumor phenotypes, such as TNBC, that rapidly progress to metastatic disease and develop treatment resistance [10]. A number of miRNAs were observed with differential expression in TNBC in comparison to non-TNBC subtypes [11]. Interestingly, as in gene expression studies, miRNA expression profiling has been shown to discriminate the intrinsic molecular breast cancer subtypes [12]. MiRNA expression varies according to ethnicity [13]. A number of studies have shown germline miRNA polymorphisms in association with the susceptibility risk of breast cancer in specific ethnic populations [14–19]. However, there are limited reports on somatic miRNA expression levels in the breast tissue of these populations [20, 21]. Consequently, the variation of the miRNA expression levels in the tumors of AA in comparison to NHW or other groups is not well known.

In this study our primary goal was to characterize the main patterns of miRNA expression in the breast tumor tissue of AA patients with TNBC in comparison to that of NHW patients with TNBC by using genome-wide miRNA profiling. A number of significant miRNAs were observed to be differentially expressed between these groups. These miRNAs were distinct from the ones differentially expressed in the non-TNBC subtypes of both AA and NHW patients. The association of the miRNA expression with copy number data, performed by array-CGH analysis in the same TNBC specimens of the AA patients, revealed a panel of 26 miRNAs which mapped in the most frequent cytobands with copy number alterations (CNAs) and with their expression levels directly corresponded to copy number gains or losses. Receiving operating characteristic (ROC) curve analysis of the individual miRNAs of this panel showed that over 65% of them presented a good discriminatory power value between the TNBC of AA and NHW patients. Subsequently, by applying a pipeline of comprehensive computational analysis, several critical and interconnected cancer gene networks and signaling pathways were found to be regulated by these miRNAs and their validated targets. Finally, association with clinical-pathological data from the patients revealed that three miRNAs from this 26 miRNA panel were associated with early patients' age at onset.

## RESULTS

### Differentially expressed miRNAs of TNBC and non-TNBC in African American and NHW women

**Figure 1:**
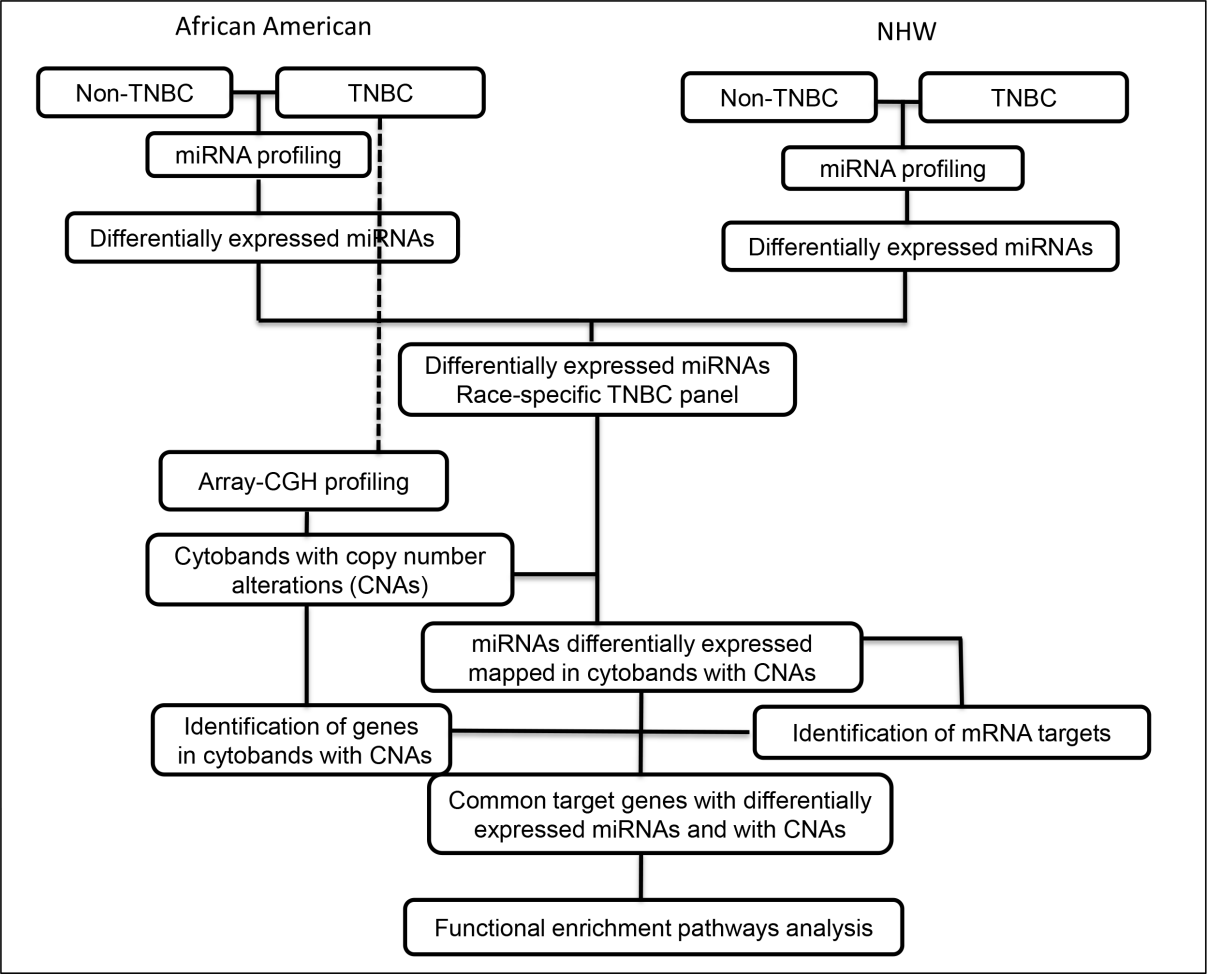
Workflow of miRNA expression and copy number profiling and downstream comprehensive computational analysis performed in the TNBC and non-TNBC cases of AA and NHW group of patients

MiRNA profiling was successfully performed in 88.9% (24/27) of the TNBC and non-TNBC (24/27) cases of the AA patients and in 93% (28/30) of the TNBC and 83.3% (25/30) of the non-TNBC cases of the NHW patients. The differentially expressed miRNAs were initially compared between each tumor subtype (TNBC and non-TNBC) for each group of patients according to the workflow of Figure 1.

**Figure 2:**
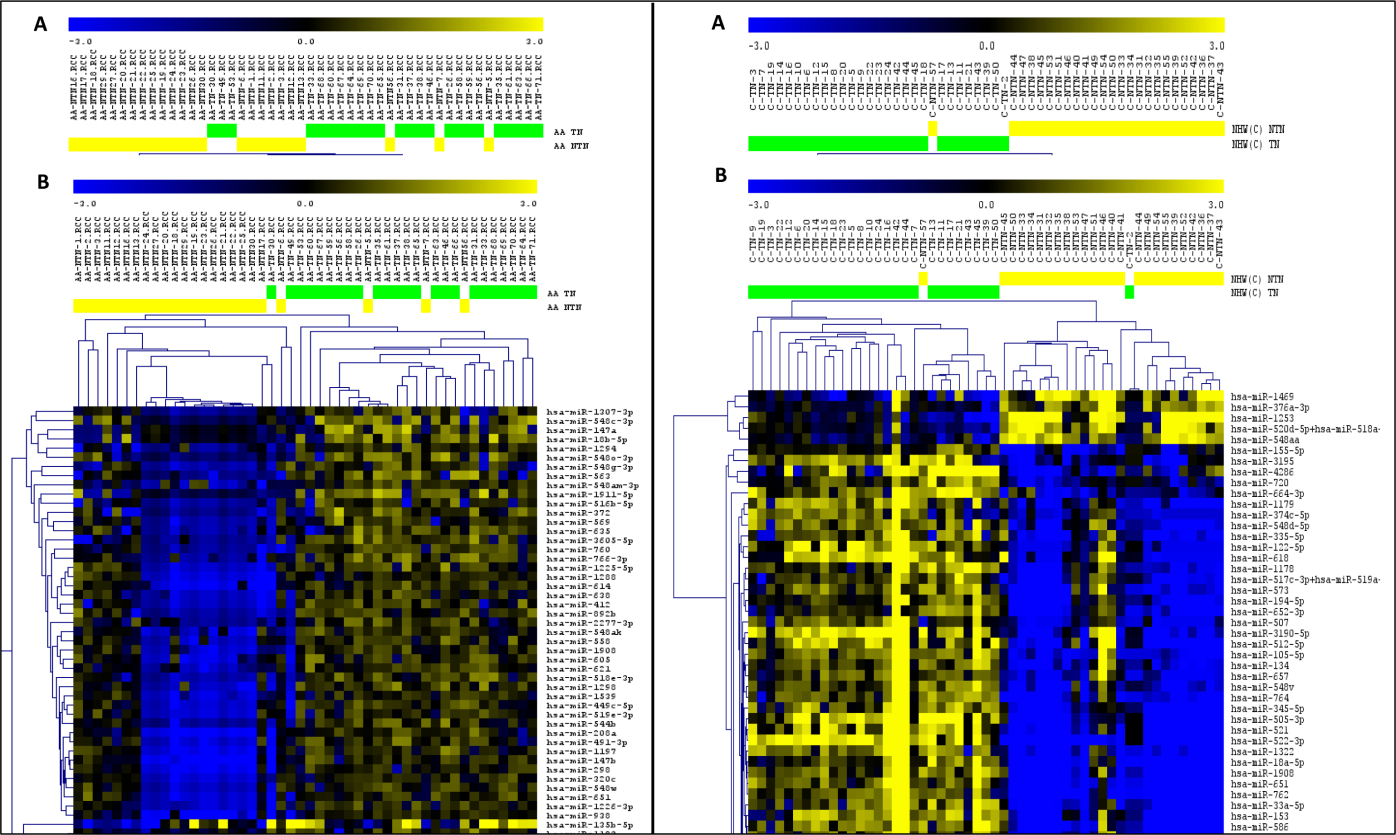
Unsupervised (A) and Supervised (B) Hierarchical Clustering analysis applied to the TNBC (green bars) and non-TNBC (yellow bars) cases of the AA and NHW group of patients (left and right panel respectively) Up-regulated miRNAs (yellow) and down-regulated miRNAs (blue). (MeV4.9; Pearson correlation, *P* < 0.01, FDR < 0.05).

The comparison of the miRNA expression levels of the TNBC and non-TNBC cases in the AA group revealed 194 miRNAs differentially expressed (*t*-test; *P* < 0.01; FDR < 0.05). The top 15 significant miRNAs observed up- and down-regulated, based on log2 fold change value, are presented in Supplementary Table S1. Unsupervised and Supervised Hierarchical Clustering analysis (Pearson correlation; *P* < 0.01, FDR < 0.05) applied to these tumors distinctly clustered the TNBC and non-TNBC tumors, with the exception of six and four cases, respectively (Figure 2).

To explore the function of each of the 194 identified miRNAs, we used DIANA miRPath analysis to perform KEGG pathway enrichment analysis. Among the top 15 pathways identified, based on *P* value, were the ones related to Pathways in Cancer, PI3K/AKT and MAPK signaling pathways (Supplementary Table S2).

The comparison of the miRNA expression levels of the TNBC and non-TNBC cases from the NHW patients revealed 336 miRNAs differentially expressed (*t*-test; *P* < 0.01; FDR < 0.05). The top 15 significant miRNAs observed up-regulated, based on log2 fold change value, are presented in Supplementary Table S3. For the down-regulated miRNAs, only a set of 12 miRNAs was observed in this analysis (Supplementary Table S3). Unsupervised and Supervised Hierarchical Clustering analysis (Pearson correlation; *P* < 0.01, FDR < 0.05) applied to these tumors distinctly clustered the TNBC and non-TNBC cases, with the exception of one and two cases, respectively (Figure 2). As for the AA group, among the top 15 KEGG pathways identified by DIANA miRPath analyses in the NHW group, based on *P* value, were the ones related to Pathways in Cancer, PI3K/AKT and MAPK signaling pathways (Supplementary Table S4).

**Figure 3:**
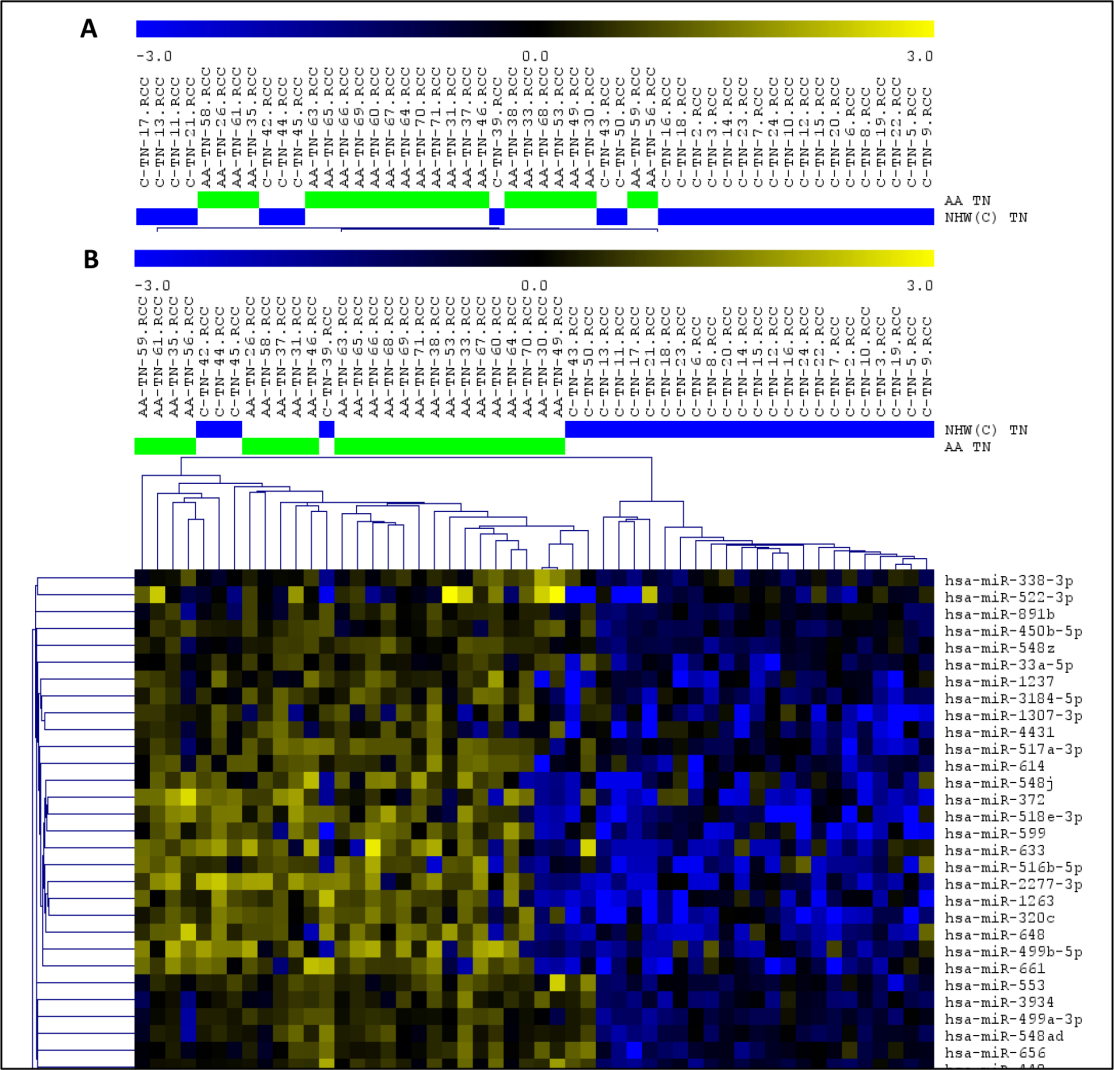
Unsupervised (A) and Supervised (B) Hierarchical Clustering analysis applied to the TNBC cases of the AA (green bars) and NHW (blue bars) group of patients Up-regulated miRNAs (yellow) and down-regulated miRNAs (blue). (MeV4.9; Pearson correlation, *P* < 0.01, FDR < 0.05).

Finally, the comparison of the miRNA expression levels of the TNBC cases from both groups of patients, revealed 256 miRNAs differentially expressed (*t*-test; *P* < 0.01, FDR < 0.05). The top 15 significant miRNAs observed up- and down-regulated, based on log2 fold change value, are presented in Table 1. Unsupervised and Supervised Hierarchical Clustering analysis (Pearson correlation; *P* < 0.01, FDR < 0.05) distinctly clustered the TNBC from the AA and NHW patients, with the exception of ten and four cases, respectively (Figure 3).

**Table 1:** Top 15 miRNAs (based on log2FC) observed up- and down-regulated in the TNBC subtype of the AA and NHW group of patients

MiRNAs up-regulated	Log2 FC	*P*-value	FDR	MiRNAs down-regulated	Log2 FC	*P*-value	FDR
hsa-miR-9-5p	3.138	8.61E-06	6.75E-05	hsa-miR-1253	−5.063	5.99E-10	5.99E-08
hsa-miR-127-3p	3.065	1.33E-07	2.87E-06	hsa-miR-1283	−4.601	9.57E-06	7.36E-05
hsa-miR-451a	2.993	1.56E-05	1.08E-04	hsa-miR-378e	−4.241	6.90E-06	5.75E-05
hsa-miR-205-5p	2.925	7.82E-05	3.86E-04	hsa-miR-549	−4.136	3.45E-08	1.25E-06
hsa-miR-548p	2.850	4.07E-08	1.36E-06	hsa-miR-1268b	−3.082	3.41E-13	2.72E-10
hsa-miR-4508	2.838	8.25E-08	2.06E-06	hsa-miR-1265	−2.781	3.80E-11	1.01E-08
hsa-miR-4425	2.829	2.36E-08	9.45E-07	hsa-miR-433	−2.750	2.22E-12	8.88E-10
hsa-miR-150-5p	2.730	6.20E-04	0.002119733	hsa-miR-1305	−2.748	1.30E-08	5.80E-07
hsa-miR-374a-5p	2.682	2.03E-05	1.33E-04	hsa-miR-518f-3p	−2.746	4.11E-11	8.23E-09
hsa-miR-455-3p	2.507	2.51E-09	2.01E-07	hsa-miR-649	−2.731	2.93E-10	3.35E-08
hsa-miR-424-5p	2.499	3.97E-07	5.78E-06	hsa-miR-520e	−2.689	4.79E-11	7.67E-09
hsa-miR-423-5p	2.467	3.15E-07	5.25E-06	hsa-miR-206	−2.671	3.38E-08	1.29E-06
hsa-miR-200c-3p	2.465	6.10E-04	0.002093624	hsa-miR-520d-5p, 518a-5p, 527	−2.647	1.46E-06	1.60E-05
hsa-miR-606	2.453	3.54E-08	1.23E-06				
hsa-miR-26a-5p	2.449	7.54E-05	3.84E-04				

FC = fold change, FDR = false discovery rate.

### DNA copy number analysis of TNBC in African American patients and association with miRNA expression levels

**Figure 4:**
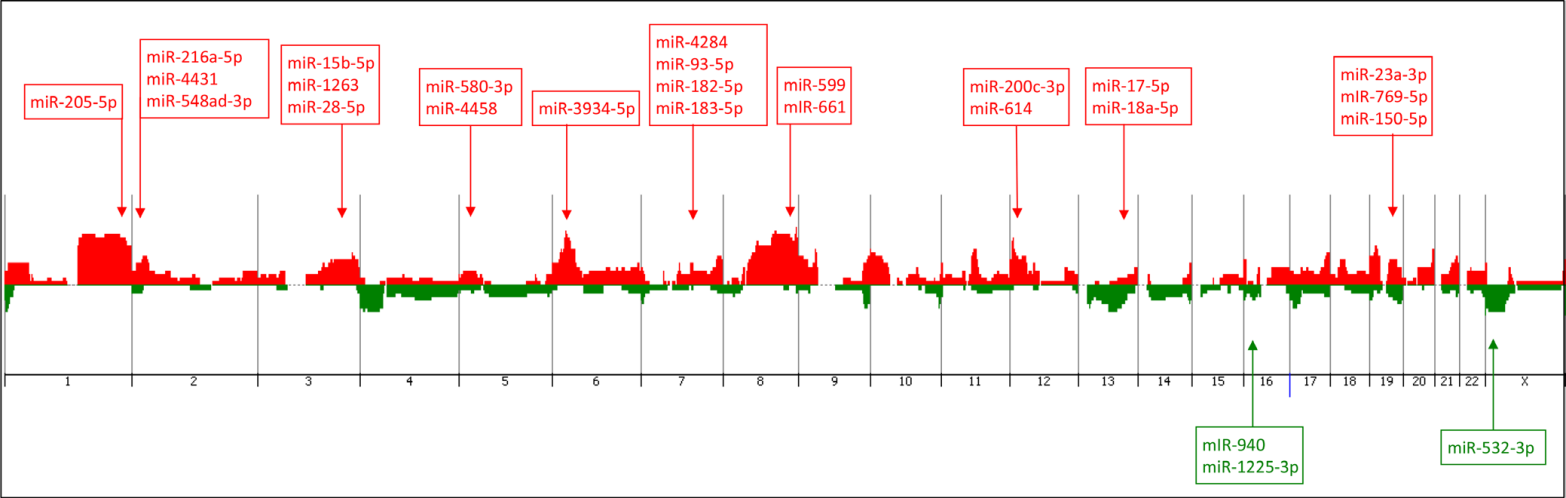
Penetrance plot of the array-CGH profiling of the TNBC cases from the AA patients analyzed, showing the corresponding genome location (arrows) of the 26 miRNAs of the identified panel Vertical lines represent each chromosome number. Red peaks indicate copy number gains and green peaks indicate copy number losses. MiRNAs with up- and down-regulated expression levels are annotated in red and green color boxes, respectively.

Array-CGH analysis was performed in the 27 TNBC cases of the AA group, 24 of which were also profiled for miRNA expression. A total number of 388 copy number alterations (CNAs) (as measured by the “number of calls”) were identified, with an average of 14.4 CNAs per case. The most frequent cytobands affected by CNAs were: 1q21.1-q44 and 8q11.1-q24 (in 55% of the cases), 3q11.1-q29 and 6p25.3-p12.1 (44% of the cases), 9p24.3-p13.1, 12p13.33-p11.1 and Xp22.33-p11.21 (39% of the cases) and 2p25.3-p11.2, 5p15.33-p12, 6q16.1-q25.3, 7q11.23-q36.3, 10p15.3-p11.1, 13q21.2-q34, 16p13.3-p11.1, 18p11.32-p11.21, 19p13.3-p12 and 19q12-q13.33 (26–33% of the cases) (Figure 4). A number of 7,362 genes were found to be located in these cytobands with CNAs, as generated by the Agilent Cytogenomics probe report.

For the association of CNAs with miRNA expression, the genomic location of the initial set of 256 miRNAs found differentially expressed between the TNBC of AA and NHW patients was verified. Fifty-five of them were located in the cytobands mostly affected by CNAs in the same AA-TNBC cases profiled by array-CGH as described above. From these 55 miRNAs, 26 presented expression levels in concordance with the observed CNAs (i.e. up-regulated miRNA expression/cytoband with copy number gain and/or down-regulated miRNA expression /cytoband with copy number loss) at their respective genome locus (Table 2, Figure 4), including the miR-150-5p, miR-200c-3p and miR-205-5p that were among the top 15 miRNAs with highest fold changes observed differentially expressed between the AA and NHW groups of patients (Table 1).

**Table 2:** Twenty-six miRNAs differentially expressed between the TNBC of the AA and NHW patients, with expression levels in concordance with copy number alterations (CNAs) (presented by chromosome numerical order)

MiRNAs	Cytoband	Start	Stop	CNAs	miRNA expression	Log2FC	*P* value
hsa-miR-205-5p	1q32.2	209432133	209432242	gain	up-regulated	2.925	7.82E-05
hsa-miR-216a-5p	2p16.1	55988950	55989059	gain	up-regulated	1.059	7.65E-07
hsa-miR-4431	2p16.2	52702522	52702615	gain	up-regulated	1.220	2.42E-04
hsa-miR-548ad-3p	2p25.1	35471405	35471486	gain	up-regulated	0.690	8.22E-04
hsa-miR-15b-5p	3q25.33	160404588	160404685	gain	up-regulated	2.202	2.97E-04
hsa-miR-1263	3q26.1	164171471	164171556	gain	up-regulated	1.375	2.69E-04
hsa-miR-28-5p	3q28	188688781	188688866	gain	up-regulated	1.655	2.28E-05
hsa-miR-580-3p	5p13.2	36147892	36147988	gain	up-regulated	1.526	7.47E-07
hsa-miR-4458	5p15.31	8460925	8460999	gain	up-regulated	2.224	4.79E-07
hsa-miR-3934-5p	6p21.31	33698128	33698234	gain	up-regulated	0.754	2.99E-04
hsa-miR-4284	7q11.23	73711317	73711397	gain	up-regulated	1.981	4.65E-06
hsa-miR-93-5p	7q22.1	100093768	100093847	gain	up-regulated	2.230	1.88E-04
hsa-miR-182-5p	7q32.2	129770383	129770492	gain	up-regulated	2.026	7.77E-05
hsa-miR-183-5p	7q32.2	129774905	129775014	gain	up-regulated	1.717	7.54E-05
hsa-miR-599	8q22.2	99536636	99536730	gain	up-regulated	1.755	7.81E-06
hsa-miR-661	8q24.3	143945191	143945279	gain	up-regulated	1.307	7.37E-04
hsa-miR-614	12p13.1	12915829	12915918	gain	up-regulated	1.021	4.31E-04
hsa-miR-200c-3p	12p13.31	6963699	6963766	gain	up-regulated	2.465	6.10E-04
hsa-miR-17-5p	13q31.3	91350605	91350688	gain	up-regulated	1.990	3.21E-04
hsa-miR-18a-5p	13q31.3	91350751	91350821	gain	up-regulated	1.160	3.60E-05
hsa-miR-940	16p13.3	2271747	2271840	loss	down-regulated	−1.118	2.05E-04
hsa-miR-1225-3p	16p13.3	2090195	2090284	loss	down-regulated	−1.953	1.29E-07
hsa-miR-23a-3p	19p13.12	13836587	13836659	gain	up-regulated	2.334	9.01E-05
hsa-miR-769-5p	19q13.32	46018932	46019049	gain	up-regulated	1.355	2.46E-06
hsa-miR-150-5p	19q13.33	49500762	49500873	gain	up-regulated	2.730	6.20E-04
hsa-miR-532-5p	Xp11.23	50003148	50003238	loss	down-regulated	−1.875	9.57E-08

CNAs = copy number alterations, FC = fold change.

**Figure 5:**
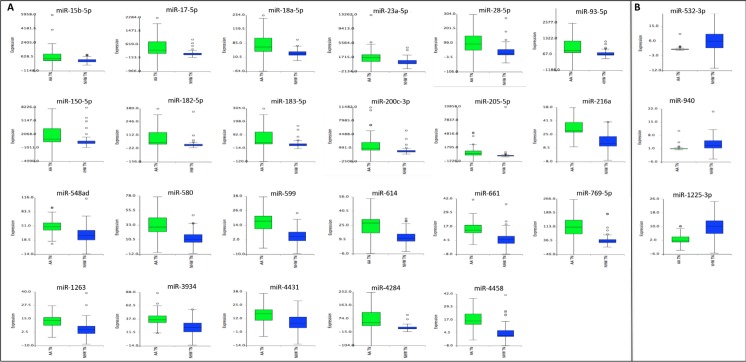
Expression levels of the 26 differentially expressed miRNAs observed between the AA and NHW TNBC cases In (**A**) and (**B**): miRNAs up-and down-regulated, respectively, in the AA group of patients when compared to the NHW group.

The average of the individual expression level (box plots) of the 26 miRNA panel in the TNBC of the AA and NHW's groups is shown in Figure 5. In the AA-TNBC group of patients, 9 to 24 of these selected 26 miRNAs were observed with alteration in their expression levels, with an average of 19.25 ± 0.85 miRNAs with expression changes per case. In the NHW-TNBC group, 8 to 24 of these miRNAs presented expression changes, with an average of 17.96 ± 0.88 miRNAs with expression changes per case. This difference was not statistically significant at *P* < 0.05.

**Figure 6:**
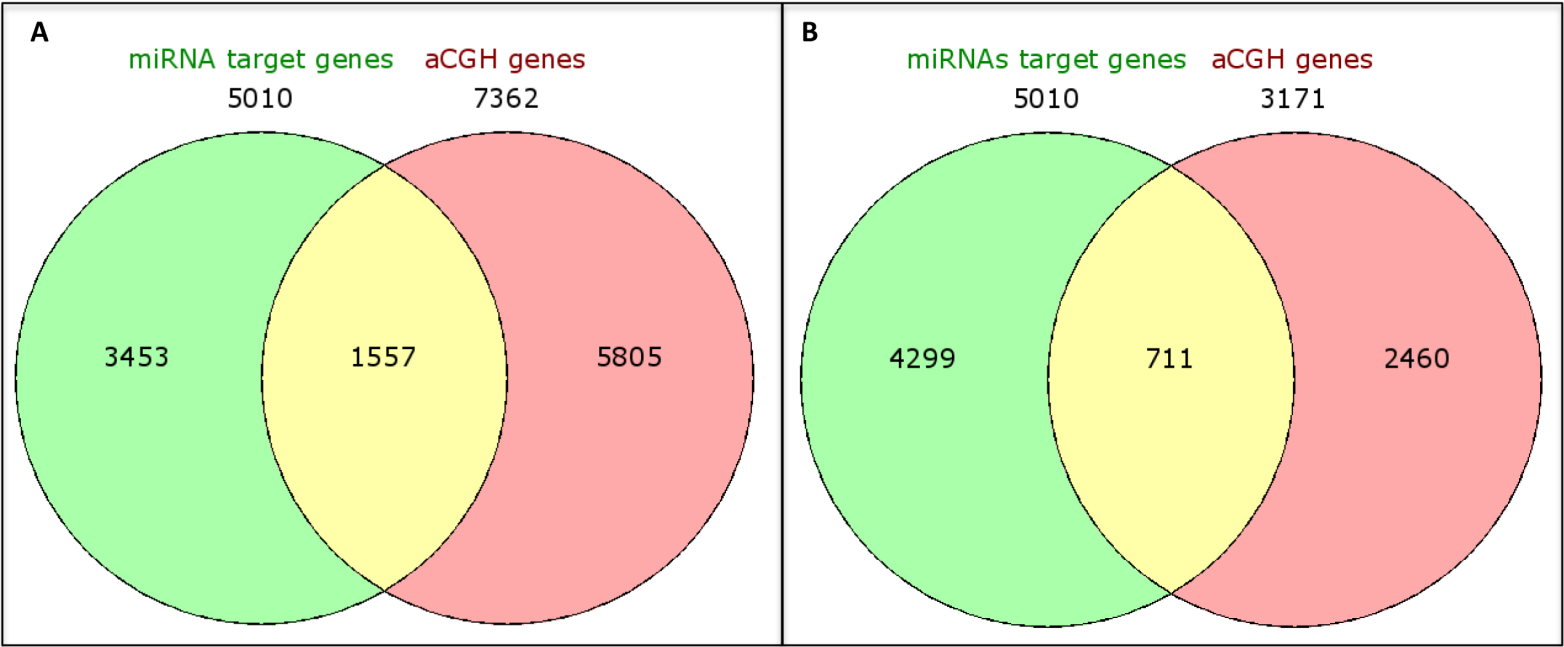
Venn diagrams showing integration of genes located at the identified cytobands (A) and in the most frequent cytobands (present in greater than or equal to 50% of the cases) (B) with CNAs in the TNBC-AA cases and the corresponding miRNA target genes

Next, we integrated the copy number and miRNA expression data to determine gene targets that were potentially affected by both of these mechanisms. The 7,362 genes identified to be located in the cytobands affected by CNAs were “matched” with the miRNA targets of the 26 selected miRNAs. A number of 5,010 miRNA targets were identified, predicted by at least two miRNA target databases. The integration of these data revealed 1,557 common genes, reducing the number of targets to 69%. However, when the miRNA targets were integrated with the genes located in the most frequent cytobands (altered in ≥ 40% of the cases: 1q21.1-q44, 3q11.1-q29, 6p25.3-p12.1 and 8q11.1-q24.3) a larger reduction (86%: from 1,557 to 711) of the targets was observed (Figure 6).

### Receiver operating characteristic (ROC) curve analysis

**Figure 7:**
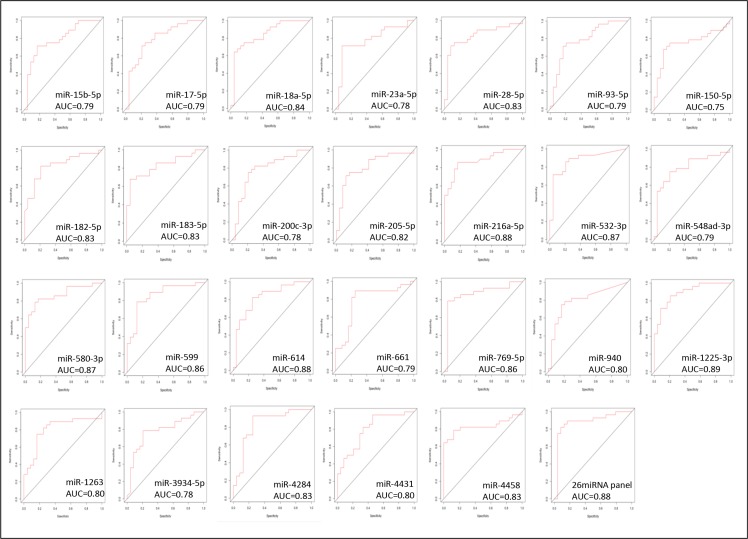
ROC plots of the individual and combined 26 miRNAs differentially expressed between the AA and NHW-TNBC group of patients

Individual ROC analysis of the 26 miRNAs of the identified panel showed that over 65% of them presented a good power in discriminating between the TNBC cases of AA and NHW group of patients [(Area Under the Curve (AUC) ≥ 0.80 with 95% Confidence Interval (CI)]. The highest discriminatory power was observed for miR-1125-3p (AUC = 0.89; 95%CI: 0.81-0.98), followed by miR-216a-5p, miR-532-5p, miR-580-3p, miR-599, miR-769-5p, miR-18a-5p, miR-28-5p, miR-182-5p, miR-183-5p, miR-1263, miR-4284, miR-4458, miR-205-5p, miR-614 and miR-940 (AUC values from 0.87 to 0.80). The remaining nine miRNAs of the 26 miRNA panel, presented AUC values ranging from 0.75 to 0.79. The combined analysis of the panel showed a AUC value of 0.88, demonstrating the robust power of the 26 miRNA panel, with a high and combined superior level of sensitivity and specificity (0.78 and 0.99, respectively) in discriminating TNBC between these populations. The ROC plots, AUC values and the corresponding 95% confidence intervals for each of the 26 miRNAs and for the combined panel are presented in Figure 7 and Supplementary Table S5, respectively.

### Functional enriched pathways

**Figure 8:**
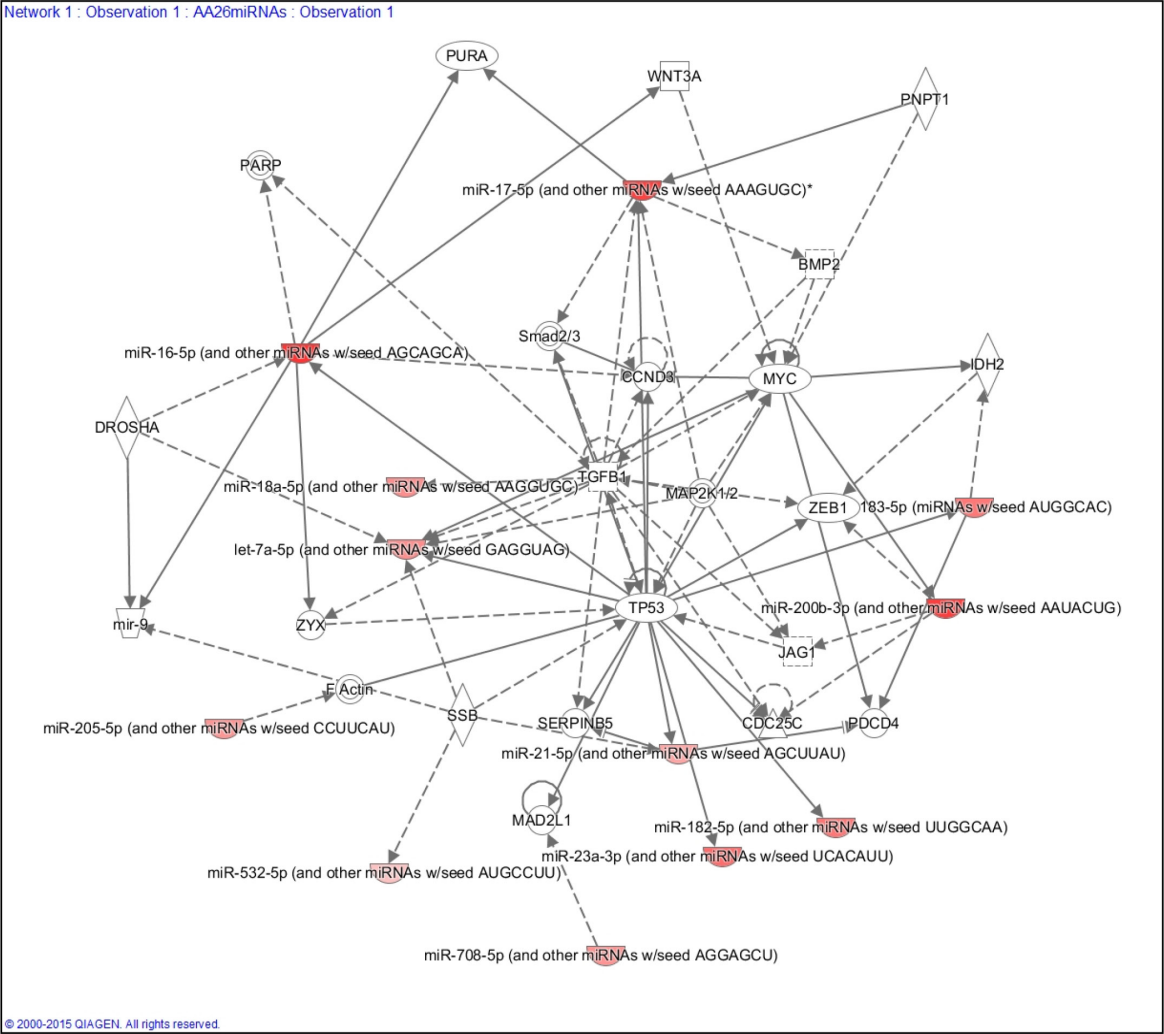
Ingenuity Pathway Analysis (IPA) showing the main gene network interaction of 12 out of the 26 miRNA panel identified

To explore the function of each of the miRNAs composing the 26 miRNA panel of differentially expressed miRNAs in the TNBC of AA and NHW groups and their corresponding targets, we performed KEGG pathway enrichment analysis. The top pathway identified (based on the lowest *P* value) was the neurotrophin signaling pathway. Twenty-three (88.5%) miRNAs of this panel were involved in this pathway. The next most significant pathways were the MAPK, insulin and PI3K/AKT signaling pathways, involving 92.3%, 88.5% and 88.5% of the 26 miRNAs, respectively. For the MAPK pathways the miRNAs not present were miR-614 and miR-4431 and for the insulin and PIK3/AKT were the miR-548ad-3p, miR-614 and miR-4431 (Table 3). These three pathways are interconnected, presenting several miRNA target genes in common (Supplementary Figure S1). IPA analysis showed that the selected 26 miRNAs are involved in the processes of cellular growth and proliferation, cell cycle, cell-to-cell signaling and interaction, and cell death and survival. This analysis also generated a gene network with 12 out of the 26 miRNAs of our panel targeting established cancer-related genes, such as *TP53*, *MYC*, *MYB*, *ZEB1*, *CCND3* and *TGFB* (Figure 8).

**Table 3:** Top 15 pathways (based on P value) mostly affected by the 26 miRNAs differentially expressed in the TNBC of AA and NHW groups of patients (DIANA miRPath v.2.0)

#	KEGG pathway	*P*-value	# Genes	# MiRNAs
1	Neurotrophin signaling pathway	7.39E-32	68	23
2	MAPK signaling pathway	1.61E-27	118	24
3	Insulin signaling pathway	1.15E-25	67	23
4	PI3K-AKT signaling pathway	3.75E-25	144	23
5	Pathways in cancer	3.75E-25	150	24
6	ERBB2 signaling pathway	1.06E-24	47	20
7	TGF-beta signaling pathway	2.75E-24	45	20
8	Focal adhesion	1.22E-23	92	23
9	Prostate cancer	3.45E-23	48	21
10	GNRH signaling pathway	5.42E-23	48	22
11	Long-term depression	1.37E-22	39	17
12	Endocytosis	1.25E-21	92	23
13	Chronic myeloid leukemia	1.09E-20	41	21
14	Renal cell carcinoma	3.06E-19	40	21
15	Axon guidance	3.26E-19	65	24

KEGG = Kyoto Encyclopedia of Genes and Genomes.

### Association of the 26 miRNA panel with the clinical-pathological variables of the TNBC and non-TNBC cases of the AA and NHW groups of patients

Clinical-pathological variables at diagnosis, such as age, tumor size, stage and grade, and lymph node metastasis status and at follow up including local and/or distant metastasis recurrence were analyzed separately for each ethnic group in the TNBC and non-TNBC subtypes (Table 4). AA patients with the TNBC subtype presented an earlier age at diagnosis when compared to patients with the non-TNBC subtype (50.74 ± 1.55 and 57.41 ± 2.79, respectively; *P* = 0.0413). AA patients with the TNBC subtype also presented a higher frequency of stage III (88.9%) and grade 3 (88.5%) tumors when compared to patients with the non-TNBC subtype (42.3% and 50%, *P* = 0.001 and *P* = 0.009, respectively). For the other clinical parameters, tumor size, lymph node, local recurrence and distant metastasis status, no significant difference was observed between the TNBC and non-TNBC cases in the AA group of patients.

**Table 4:** Analysis of clinical-pathological parameters of the TNBC and non-TNBC cases in the AA and NHW groups of patients

	AA			NHW			AA and NHW	
	TNBC	Non-TNBC	*P* value	TNBC	Non-TNBC	*P* value	TNBC	Non-TNBC
Mean age (yrs)	50.74 ± 1.549, *n* = 27	57.41 ± 2.785, *n* = 27	*P* = 0.0413*	53.70 ± 2.089, *n* = 30	53.93 ± 2.498, *n* = 30	*P* = 0.4358	*P* = 0.2687	*P* = 0.3557
Tumor size (cm)	2.819 ± 0.3821, *n* = 26	2.800 ± 0.4553, *n* = 27	*P* > 0.9999	2.964 ± 0.3074, *n* = 28	1.988 ± 0.2525, *n* = 26	*P* = 0.0185*	*P* = 0.7671	*P* = 0.1291
Tumor stage I	0	11.5% (3/26)	*P* = 0.0014**	0	27.3% (6/22)	*P* = 0.0002***	*P* = 1.0	*P* = 0.3765
Tumor stage II	11.1% (3/27)	46.2% (12/26)		10.3% (3/29)	36.35% (8/22)			
Tumor stage III	88.9% (24/27)	42.3% (11/26)		89.7% (26/29)	36.35% (8/22)			
Tumor grade 1	0	11.5% (3/26)	*P* = 0.0085**	0	27.3% (6/22)	*P* < 0.0001****	*P* = 0.6633	
Tumor grade 2	11.5% (3/26)	38.5% (10/26)		7.1% (2/28)	36.35% (8/22)			
Tumor grade 3	88.5% (23/26)	50% (13/26)		92.9% (26/28)	36.35% (8/22)			
LN- positive	50% (13/26)	63.2% (12/19)	*P* = 0.5446	38.5% (10/26)	56.3% (9/16)	*P* = 0.3437	*P* = 0.5771	*P* = 0.7391
LN- negative	50% (13/26)	36.8% (7/19)		61.5% (16/26)	43.7% (7/16)			
BC rec-positive	33.3% (9/27)	11.1% (3/27)	*P* = 0.0994	10.3% (3/29)	3.8% (1/26)	*P* = 0.6131	*P* = 0.0519*	*P* = 0.604
BC rec-negative	66.7% (18/27)	88.9% (24/27)		89.7% (26/29)	96.2% (25/26)			
DM-positive	25.9% (7/27)	19.2% (5/26)	*P* = 0.7445	17.2% (5/29)	19.2% (5/26)	*P* = 1.0	*P* = 0.5225	*P* = 1.0
DM-negative	74.1% (20/27)	80.8% (21/26)		82.8% (24/29)	80.8% (21/26)			

AA = African American, NHW = Non Hispanic Whites, TNBC = triple negative breast cancer, LN = Lymph Node, BC rec = Breast Cancer recurrence, DM = distant metastasis.

In the NHW group of patients, no difference was observed in the mean age at diagnosis between the TNBC and non-TNBC subtypes. However, tumor size was significantly higher in the TNBC subtype of the NHW patients when compared to the non-TNBC subtype (2.96 ± 0.31 and 1.99 ± 0.25, respectively; *P* = 0.019). A higher level of significance was observed regarding tumor stage and grade in this group of patients: TNBC patients presented 89.7% of stage III and 92.8% of grade 3 tumors when compared to the non-TNBC patients (36.4% of both stage III and grade 3 tumors; *P* = 0.0002 and *P* < 0.0001 respectively). As for the AA patients, the other clinical parameters evaluated were not differently distributed in the tumor subtypes of the NHW patients.

Next, these analyses were performed comparing the tumor subtypes between the AA and NHW groups. A “borderline” significant difference was observed in relation to the presence of local recurrence in the TNBC group; AA patients presented a higher frequency of local recurrence when compared to the NHW patients (33.3% and 10.3%, respectively; *P* = 0.0519). None of the other parameters were significantly different in the TNBC or non-TNBC subtypes between these patients' groups (Table 4).

The association of the expression levels of the 26 miRNA panel and clinical-pathological variables from the TNBC patients revealed that three miRNAs were significantly associated with age at the time of surgery, while adjusting for ethnicity, tumor size, and lymph node status: miR-216a-5p, miR-580-3p and miR-4458 (adjusted *P* value = 0.026 for all three). They all showed a positive association with age. Neither tumor size nor lymph node status was significantly associated with the expression levels of any of the 26 miRNAs evaluated in this analysis. This analysis excluded 7 samples which had missing clinical-pathological parameters.

## DISCUSSION

The triple negative breast cancer (TNBC) subtype is observed with higher frequency in African American (AA) women in comparison to non-Hispanic White (NHW) women, usually in association with poor prognosis and high mortality rate [4, 5, 8]. This disparity has been attributed to several causes, including tumor characteristics at diagnosis, such as age, tumor size, stage and grade [8]. In our study, however, none of these factors were significantly different between the AA and NHW' patients studied; the same occurred for the non-TNBC subtypes between the groups. These findings were in agreement with previous studies showing that age and pathology stage were not different between these populations, specially in the TNBC subtype [8]. However, within each group of patients we did observe significant differences at these parameters. In the AA group, a younger age at diagnosis, higher frequency of advanced tumor stage and poorly differentiated tumors were observed in the TNBC when compared to the non-TNBC subtypes. In the NHW group the same was observed for tumor grade and stage, but not for age at diagnosis, which is in agreement with several other reports, showing that age at diagnosis has a higher impact in the AA population than in other populations [5].

MiRNA expression has been shown to present an extraordinary power in classifying breast tumor subtypes [12]. In this study, genome-wide miRNA profiling distinctively clustered most of the TNBC and non-TNBC subtypes of both AA and NHW patients. In the NHW group only two (3.8%) cases were “misclassified” by the miRNA profiling (336 miRNAs differentially expressed) as opposed to four (8.3%) cases in the AA group (194 miRNAs differentially expressed). These results confirm the robust power of miRNA profiling in differentiating the intrinsic breast cancer molecular subtypes.

One of the most challenging aspects of analyzing tumor cells using whole genome miRNA platforms is the selection and “triage” of the most biologically relevant miRNAs and their corresponding targets among the large output data generated by these high-density platforms. One of the strategies utilized includes multi-platforms integration and functional enriched pathway analysis [21]. MiRNAs are frequently located in regions of genomic instability, markedly characterized by the presence of gains and losses of genomic regions [22–24]. Previous studies have integrated the data from miRNA expression and copy number profiling in human tumors [25–27]. Most of these studies were however, not performed in tumor tissue specimens that were concomitantly analyzed by these methods; in fact the vast majority of them were based on the miRNA and array-CGH profiling data extracted from public genomic datasets [26, 27], which invariably introduce a high level of technical and sample heterogeneity considering that array platforms with different annotations and variable sample sources and material are “uniformly” combined.

In our study, these technical issues were eliminated, given that both analyses were performed in the same tissue specimens. This strategy allowed for the direct mapping of the total initial number of miRNAs differentially expressed in the TNBC of AA and NHW patients, in the genomic regions carrying CNAs, from which 26 miRNAs presented expression levels directly corresponding to copy number gains or losses. These findings are in agreement with the integration analysis performed by others, where tumor' miRNAs were located in genomic regions frequently amplified and/or deleted in cancer [22–24]. It is relevant to point out, however, that because some of the regions with genomics gains and losses are defined by large cytobands, it is not unusual to find the same region with either gain or loss of copy number, which can explain some of the lack of concordance of the miRNA expression levels mapped on these genomic regions [24]. In addition, several other mechanisms can impact miRNA expression regulation other than CNAs [28].

However, among the 26 miRNAs of our study, fourteen (miRs 205-5p, 15b-5p, 1263, 28-5p, 4284, 93-5p, 182-5p, 183-5p, 599, 661, 200c-3p, 17-5p, 18a-5p and 23a-3p) were located in cytobands frequently amplified in TNBC [20, 29], including the 1q21.1-q44, 3q11.1-q29, 7q11.23-q36.3, 8q11.1-q24.3, 12p13, 13q21.2-q34, and 19p13, respectively. The miRs 200c-3p, 205-5p, 548ad-3p, 661, 17-5p, 18a-5p and 93-5p which were among the top 15 miRNAs with highest fold changes observed differentially expressed between the AA and NHW groups, are mapped in these locations (12p13, 1q32.2, 1q32.2, 8q23-24, and 13q31.3, 13q31.3 and 7q22.1, respectively). These miRNAs have been previously described with deregulated expression levels in breast cancer in association with cancer related pathways such as the ones involved in epithelial mesenchymal transition (EMT), cell migration, invasion and treatment resistance [30–33]. In addition, ROC curve analysis of the individual 26 miRNAs showed for 65.4% of these miRNAs a good discriminatory power in discriminating TNBC of AA and NHW patients. The ROC curve analysis of the combined miRNAs (AUC of 0.88) showed a superior power in discriminating these two populations, supporting the overall robustness of this panel. These results indicate that these 26 miRNAs are not randomly affected in TNBC, and may constitute significant differences in the biology of TNBC in these populations.

MiRNA expression levels have been demonstrated to vary according to ethnicity [13]. Several studies have shown the presence of genetic variants, mostly of single nucleotide polymorphisms (SNPs) in miRNA sites in association with the susceptibility risk of breast cancer in specific ethnic populations [14–19]. While in this study, we reported on “somatic” miRNA expression levels among the tumor tissue of the AA and NHW populations, which is not as frequently described, it is important to consider that the presence of these polymorphisms can modify miRNA expression, which could imply that some of the differences noted were not necessarily related to the tumor etiology, but rather to population stratification. Therefore, we queried the available miRNAs-SNPs databases (former HapMap [34], 1000 Genome Project [35]) and other reports on miRNA-SNPs in human populations [13, 15, 19], which include datasets of Lymphoblastoid cell lines (LCLs) derived from CEU (Utah residents with northern and western European ancestry) and YRI (Yoruba people from Ibadan, Nigeria) [34] for the presence of these genetic variants, that could affect the expression of the miRNAs composing our panel in the populations studied. Although SNPs were reported enriched in the AA and/or White population in few of the miRNAs of our 26 miRNA panel, e.g. miR-183-5p, miR-661, they were mostly present in the seeding sequences of the miRNAs (3′UTR sequences), which could present a higher impact in the impairment of their interaction with their corresponding targets and functional activity, rather than their expression levels. We evaluated by qRT-PCR the miRNA expression of few miRNAs composing our panel, including the miR-661, in the normal breast tissue from a subset of the AA and NHW breast cancer patients of this study (data not shown) and did not observed the altered expression levels that was shown in the tumor, ruling out this possible polymorphism “effect” in their expression levels.

The pathway and function enrichment analysis of the 26 miRNAs differentially expressed in TNBC of AA and NHW of our study, confirmed their association with breast cancer tumorigenic processes, especially the ones that confer clinically aggressive tumor phenotypes, such as the ones in TNBC.

The miR-205-5p in particular, have been described down-regulated in the TNBC subtype, which is in alignment with its tumor suppressor role in the inhibition of proliferation, migration and invasion of cancer cells [36, 37]; others, as in our study, have reported its up-regulation, compatible with its oncogenic role in tumor initiation and proliferation [38]. Due to its differential expression in serum among cancer patients and healthy individuals, this miRNA has been considered a new biomarker for early detection of cancer [39, 40].

MiR-599 and miR-661, which were up-regulated in the TNBC cases of the AA group of this study, are mapped in cytobands with high level of amplification, the 8q23-24 region, which is often associated with basal tumors that present with focal amplification of the *C-MYC* oncogene [41, 42]. Although this is a region frequently affected in breast cancer irrespective of the molecular subtype and ethnicity [20], it has been observed preferentially amplified in TNBC, including the ones that present *BRCA1* mutations [43], which are frequently in AA women [44, 45].

Two clusters of miRNAs in our study were previously observed up-regulated in TNBC, with copy number directly influencing expression levels [25]; the cluster comprising miR-17-5p and miR-18a-5p, and the cluster of miR-93-5p, both located in 13q31.3, a commonly amplified genomic region in the TNBC of the AA patients. Targets of these miRNAs clusters are involved in gene networks associated with tumor aggressiveness, including the ones involving *PIK3CA*, *C-MYC* and *PTEN* genes [46–51]. In fact, the identification of the main pathways and gene networks affected by the corresponding miRNA targets of our 26 miRNA panel supported these reported findings. The neurotrophin signaling pathway was the one mostly affected, followed by the interconnected PI3K/AKT, MAPK kinase and insulin pathways. With the exception of three miRNAs, miR-548ad-3p, miR-614 and miR-4431, 23 miRNAs in our panel were involved in at least one of these pathways. Growth factors of the neurotrophin family and their receptors have been shown to be involved in breast cancer, affecting tumor cell growth and metastasis [52–54]. PI3K/AKT, MAPK kinase and insulin pathways are frequently described in breast cancer, which downstream targets control cell proliferation, cell survival and glucose metabolism [55].

The target pathways of our study were also among the most common ones observed in the TNBC cases of the TCGA and other studies [20, 56–58], with downstream genes involved in the *VEGF*, *C-MYC* and *PIK3* gene networks. In the TCGA study [20], the *PIK3* network presented with the highest levels of gene deregulations, involving critical downstream genes, such as *PTEN*, one of the most relevant mRNA targets of our 26 miRNA panel. In fact, our previous study of TNBC in Latina women, also suffering from TNBC disparities, showed a high frequency of *PTEN* loss (62%) in significant association with advanced tumor grades [59]. Interestingly, the insulin pathway, which has also been shown to be regulated by miRNAs [60], including the miR-1225-5p in our study [61], was among the top pathways of the differentially expressed miRNAs in the TNBC and non-TNBC subtypes of the AA patients, but not in the NHW group of patients. These findings are supported by previous reports showing differential expression of the Insulin Growth Factor (*IGF-1*) gene and its receptors in association with breast cancer subtypes and ethnicity [62, 63]. In AA patients in particular, alterations in this pathway and its downstream targets have been shown to contribute to the increased risk of malignant transformation in young women and to confer more aggressive breast cancer subtypes [56–58]. Consistent with our findings, previous studies have shown that the transcriptional profile of the TNBC basal 1 subtype, which is often the most frequent TNBC subtype in AA [64], was associated with a low IGF1 signature score, a marker of high IGF1 receptor (IGF1R) expression and up regulation of the MAPK and AKT growth pathways [56]. A number of IGF1 receptor inhibitors are currently in preclinical and clinical trials [65, 66]; our and others data suggest that inhibitors of the IGF1 pathway may be a sensitive target in TNBC, particularly benefiting AA patients.

Interestingly, in our study, the insulin pathway was not specifically associated with the TNBC subtype in the AA patients, which indicates its relevance in the etiology of breast cancer in general in this population, which may be directly correlated with the high incidence of obesity and other co-morbidities, such as diabetes and other metabolic syndromes in this population [67]. Considering the critical role of the identified miRNAs in directing regulating targets associated with these co-morbidities, overall, these findings can form the basis to build a race specific genomic signature associated with co-morbidities, which can promote and augment prevention and intervention strategies, stratify and select appropriate patients for treatment and clinical trials and personalize cancer care.

In conclusion, we observed different patterns of miRNA expression in the TNBC of AA and NHW women in this study. The 26 miRNA panel observed in association with CNAs in the AA patients, presented a high power in discriminating TNBC between AA and NHW patients and affected critical cancer related gene networks and signaling pathways. These findings indicate that the mapping of the miRNAs in genomic regions with high levels of CNAs of this study was not merely physical, but biologically relevant to the TNBC genome of AA patients. The validation of this 26 miRNA panel in independent and larger samples sets from these ethnic groups is required to ensure that their expression patterns are reflected across populations and are not unique to a particular cohort of patients. The future determination of their functional regulatory role in conferring the aggressive TNBC phenotype, including early development of metastasis and drug resistance, can open new opportunities to develop novel therapeutic targets for TNBC, holding promises towards the improvement of the overall survival rate of AA women with TNBC.

## MATERIALS AND METHODS

### General study design

Genome-wide miRNA profiling was performed in TNBC and non-TNBC cases from AA and NHW patients. The array-CGH analysis performed in the same TNBC specimens of the AA patients was associated with the miRNA expression profiling data as presented in the workflow of Figure 1. ROC analysis was performed to determine the individual discriminatory power of the identified panel of miRNAs in the TNBC of the studied populations. The identified miRNAs and corresponding targets were then biologically selected for their relevance in the breast cancer/TNBC phenotype by applying a comprehensive computational analysis using combinatorial target prediction algorithms in conjunction with Gene Ontology and pathway enrichment analysis. Finally association with clinical-pathological data from the patients was performed.

### Patient accrual and sample collection

Formalin-fixed paraffin-embedded (FFPE) tumor sections were obtained from 27 and 30 cases of TNBC from AA and NHW breast cancer patients, respectively, from patients that undergone primary surgery for tumor removal, prior to any treatment, at the MedStar Georgetown Hospital, Washington DC. Tumors with a non-TNBC subtype were also obtained from both groups in the same number of patients: 27 non-TNBC from AA and 30 non-TNBC cases from NHW patients. Altogether 54 AA and 60 NHW patients were evaluated in this study. All the samples were procured from the patients (from all groups of tumors (TNBC and non-TNBC) and ethnic groups (AA and NHW) under informed consent, performed by the personal from Non-Therapeutic Shared Resource (NTSR) of Lombardi Comprehensive Cancer Center according to their establish SOP. The specimens were received de-codified, with no patient identifiers, under the Histopathology Tissue Shared Resources (HTSR)-IRB approved protocol (IRB#1992-048).

Clinical and pathological information was retrieved by the HTSR personnel and included: age at diagnosis, tumor size, stage and grade, and presence of lymph node metastasis. Breast cancer recurrence and distant metastasis status were also obtained with a follow-up period that varied from 2 to 9 years (Table 4). The analyzed breast cancer tissues were from primary tumor lesions, obtained prior to any cancer treatment, at the time of the surgery. The classification of the breast cancer TNBC and non-TNBC phenotype was determined by the three IHC surrogate markers ER, PR and HER2, following the American Society of Clinical Oncology (ASCO)/College of American Pathology (CAP) guidelines [68, 69]. In the non-TNBC group, 65% of the cases were ER+/PR+/HER2-, followed by 19% of ER+/PR+/HER2+, when combining AA and NHW patients. Prior to the genomic analysis all of the tumor tissues were inspected for the presence of > 80% of tumor cells followed by microdissection according to a previous protocol [70].

### Ancestral markers analysis

Ethnicity information was primarily obtained from self-reported patients' records. To obtain a genomic based information and assess ancestry we genotyped a subset of patients (13 AA and 14 NHW) using SNP chip Illumina Infinium QC Array (Illumina Inc., CA), which contains 15,949 markers (including ~3,000 ancestral informative markers (AIMs). The genotype calling was performed using GenomeStudio Software v. 2011.1 using the default settings. SNPs with MAF ≤ 0.01 were excluded from analysis. Further, we merged our dataset with the 1000 Genomes Project phase 1 (*n* =1,902 samples) [71], which present an overlap of 14,718 variants between ours and these data sets. Finally, we performed Principal Components Analysis (PCA) using PLINK 1.9 [72], which uses the EIGENSTRAT method [73] to calculate model ancestry differences between different samples. Based on the result of PC1 and PC2 we were able to differentiate the two main population groups (European (EUR) and African (AFR) in our samples confirming the self-report ethnicity information (Supplementary Figure S2).

### MiRNA analysis

MiRNA expression analysis was performed using the Human v2 miRNA Expression Assay from NanoString nCounter Technology (Seattle, WA, USA), that contains 800 human probes derived from miRBase v.18. The raw data was pre-processed by NanoString's nCounter RCC collector and the miRNAs were normalized using the geometric mean. Unsupervised and supervised hierarchical cluster analysis was performed on miRNAs that were found to be significantly differentially expressed (*P* < 0.01, FDR < 0.05), using Pearson's correlation coefficient and average linkage by using the Multiexperiment Viewer software (MeV 4.9.0). Fold changes, represented on the log2 scale (log2FC), were calculated for all differentially expressed miRNAs.

### Array-CGH analysis

DNA copy number analysis was performed using an oligonucleotide array-CGH platform (SurePrint G3 Human CGH Microarray 8×60K; Agilent Technologies Inc., Santa Clara, CA), according to the protocol for FFPE samples that we have established in our lab [70]. DNA was isolated from consecutive FFPE sections of the cases profiled for miRNA. DNA isolated from peripheral blood from multiple normal individuals was used as control DNA. The array data was analyzed using the Feature Extraction (FE) v.10.10 and Agilent CGH Analytics v.7.0 software (Agilent Technologies Inc., Santa Clara, CA), using the ADM-2 algorithm, threshold 6.0 and an aberration filter with a minimum number > 3 probes. Gene amplifications and deletions were defined as minimum average absolute log2 ratio (intensity of the Cy5 dye (reference DNA)/intensity of the Cy3 dye (test DNA) value of > 0.25 and <−0.25, respectively, as per the CGH analytics analysis.

### Association of miRNA expression levels and copy number alterations (CNAs)

To determine the potential association of miRNA expression levels and CNAs, we integrated the miRNA and copy number data that was performed in the same TNBC tissue specimens of the AA patients. Initially, the genomic location of each miRNA differentially expressed between the TNBC of AA and NHW patients was assessed at the miRbase.org and determined whether they were mapped at the most commonly affected cytobands (CNAs present in ≥ 33% of the cases). Next, it was determined whether the miRNAs residing in these cytobands presented the corresponding changes in expression levels (i.e. cytoband with copy number gain/up-regulated miRNA expression and cytoband with copy number loss/down-regulated miRNA expression). A second integration approach was to determine the common gene targets that may be affected by both CNAs and miRNA expression alterations. For this approach, gene targets were queried using the available miRNA target databases and integrated with the genes located in the most commonly cytobands above. Only miRNA target genes that were present in two out of the three miRNA databases were selected. Considering that one miRNA can target several different genes irrespectively of their genomic location, all of the most frequently affected cytobands (present in at least 33% of the TNBC-AA cases) were included in this analysis.

### Receiver operating characteristic (ROC) curve analysis

The ability of the identified 26 miRNA panel to discriminate between AA-TNBC and NHW-TNBC was examined by constructing receiver operating characteristic (ROC) curves and calculating the area under the curve (AUC). Sensitivity was plotted against 1-specificity for the binary classifier (AA-TNBC and NHW-TNBC). An AUC of 100% denotes perfect discrimination by the miRNA, whereas an AUC of 50% denotes complete lack of discrimination by the miRNA. AUCs and 95% corresponding confidence intervals were calculated for each miRNA.

### Biological function and pathway analysis

The targeted pathways of the identified miRNAs were determined by DIANA miRPath v.2.0 software [74]. A detailed functional analysis to identify miRNA-mediated, cancer-related and statistically significant networks, biological functions and canonical signaling pathways for both differentially expressed miRNAs and target genes was performed by Ingenuity Pathway Analysis System (IPA v.8.5, Ingenuity Systems, Edwood, CA).

### Analysis of clinical-pathological variables of the TNBC and non-TNBC of AA and NHW patients

The Student *t* test was used to analyze the differences of the mean age at diagnosis and tumor size in the TNBC and non-TNBC cases within and between the patient groups. The Chi-square (χ^2^) test was used to evaluate tumor stage and grade and the Fisher Exact test was used to evaluate lymph node, local recurrence, and distant metastasis status in both tumor subtypes. Significance level was considered to be *P* < 0.05. For the 26 miRNAs that were selected from the integration with the array-CGH data, linear regression models were considered having the transformed miRNA values as the outcomes and ethnicity, age, tumor size, and lymph node status as the regressors. For each of the clinical-pathological parameters (age, tumor size, lymph node status), a significance level of FDR < 0.05 using the Benjamin and Hochberg FDR control method [75] was considered. Tumor stage and grade were not considered since none of the AA and NHW patients with the TNBC subtype showed tumors with stage I and grade 1.

## SUPPLEMENTARY MATERIALS


