# Supplementary material for: Differentially expressed miRNAs in triple negative breast cancer between African-American and non-Hispanic white women
Source: Oncotarget. 2016 Nov 2;7(48):79274–91. doi: 10.18632/oncotarget.13024 (PMC5346713; doi:10.18632/oncotarget.13024)
Supplement: Supplementary file 1 [file oncotarget-07-79274-s001.pdf]

# Differentially expressed miRNAs in triple negative breast cancer between African-American and non-Hispanic white women

## Supplementary Materials

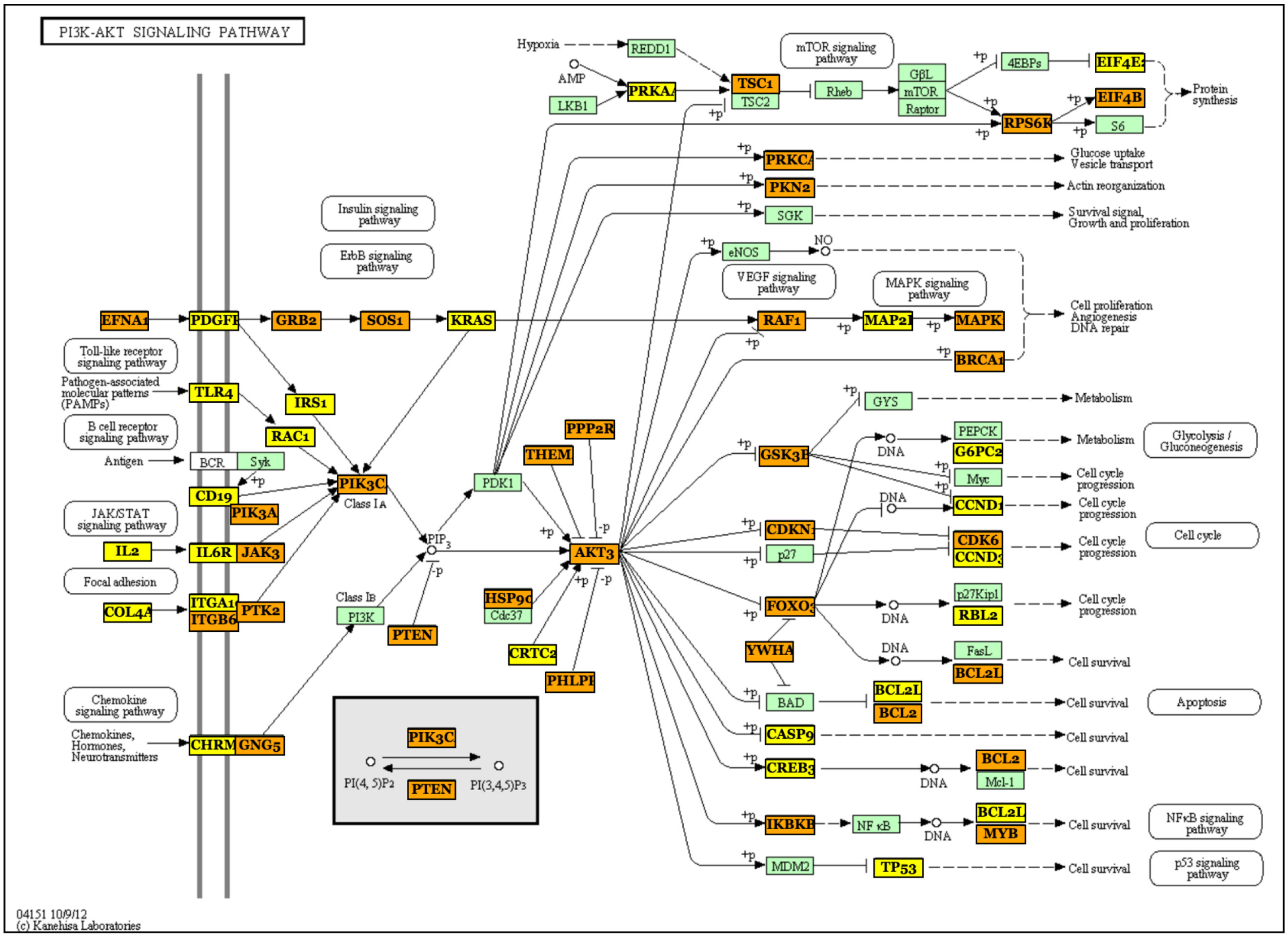


**Supplementary Figure S1: PIK3-AKT and other signaling related pathways affected by the 26 miRNAs of our panel (DIANA miRPath v.2.0).** White boxes indicate gene entries in non-metabolic pathways or enzyme and reaction entries in metabolic pathways (Reference Pathway). Green boxes indicate presence of the gene in the genome (Organism-specific pathway). Yellow boxes indicate genes targeted by one miRNA. Orange boxes indicate genes targeted by two or more miRNAs.

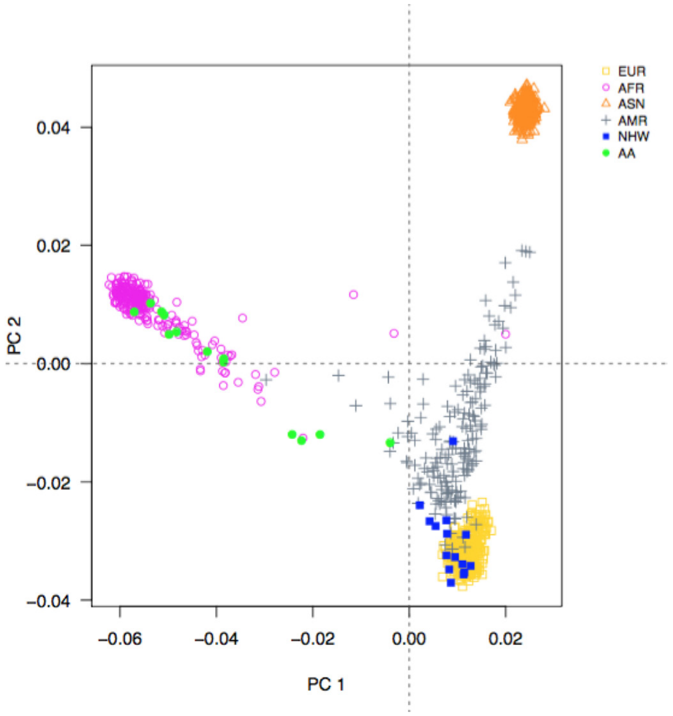


**Supplementary Figure S2: Principal component analysis (PCA) showing that a subset genotype dataset of African American (AA) and Non-Hispanic White (NHW) patients of this study, distinctly clustered with the European (EUR) and African (AFR) main ethnic groups from the 1000 Genome Project (phase 1).** ASN: East Asian, AMR: Latin American.

## Supplementary Table S1: Top 15 miRNAs (based on log2FC) observed up- and down-regulated in the TNBC subtype in comparison to the non-TNBC subtype of the AA group of patients

| MiRNAs up-regulated | Log2 FC | *P*-value | FDR | MiRNAs down-regulated | Log2 FC | *P*-value | FDR |
|---|---|---|---|---|---|---|---|
| hsa-miR-384 | 2.559 | 4.05E-05 | 7.20E-04 | hsa-miR-497-5p | –4.079 | 1.89E-08 | 1.51E-05 |
| hsa-miR-135b-5p | 2.360 | 9.31E-04 | 0.004433157 | hsa-miR-145-5p | –3.251 | 1.28E-06 | 1.28E-04 |
| hsa-miR-1277-3p | 2.358 | 5.79E-05 | 8.91E-04 | hsa-miR-29c-3p | –3.105 | 1.75E-07 | 7.00E-05 |
| hsa-miR-1185-5p | 2.304 | 7.74E-06 | 3.10E-04 | hsa-miR-143-3p | –3.036 | 1.47E-06 | 1.31E-04 |
| hsa-miR-626 | 2.245 | 2.08E-06 | 1.19E-04 | hsa-miR-26a-5p | –2.976 | 3.20E-06 | 1.70E-04 |
| hsa-miR-219-5p | 2.194 | 7.87E-06 | 3.00E-04 | hsa-miR-1253 | –2.879 | 4.51E-04 | 0.0027133 |
| hsa-miR-576-5p | 2.190 | 1.69E-06 | 1.23E-04 | hsa-miR-199b-5p | –2.825 | 1.65E-06 | 1.32E-04 |
| hsa-miR-606 | 2.164 | 2.79E-07 | 7.43E-05 | hsa-miR-342-3p | –2.785 | 4.42E-07 | 7.07E-05 |
| hsa-miR-499a-3p | 2.132 | 4.86E-05 | 7.94E-04 | hsa-miR-1290 | –2.763 | 7.36E-06 | 3.10E-04 |
| hsa-miR-548al | 2.093 | 8.52E-06 | 2.96E-04 | hsa-miR-29b-3p | –2.742 | 8.27E-05 | 0.001050187 |
| hsa-miR-522-3p | 2.091 | 1.72E-06 | 1.15E-04 | hsa-miR-30a-5p | –2.720 | 1.41E-04 | 0.001429822 |
| hsa-miR-516a-5p | 2.029 | 7.06E-05 | 9.57E-04 | hsa-miR-196a-5p | –2.668 | 4.62E-05 | 7.69E-04 |
| hsa-miR-659-3p | 2.020 | 4.01E-05 | 7.29E-04 | hsa-miR-195-5p | –2.631 | 3.72E-07 | 7.45E-05 |
| hsa-miR-568 | 2.016 | 1.43E-05 | 4.08E-04 | hsa-miR-451a | –2.613 | 8.64E-05 | 0.001063546 |
| hsa-miR-518c-3p | 1.993 | 1.91E-05 | 4.50E-04 | hsa-miR-30b-5p | –2.555 | 3.15E-05 | 6.45E-04 |

FC = fold change, FDR = false discovery rate.

**Supplementary Table S2: Top 15 KEGG pathways (based on P value) mostly affected by the differentially expressed miRNAs in the TNBC and non-TNBC subtypes of the AA group of patients (DIANA miRPath v.2.0)**

| # | KEGG pathway | *P*-value | # Genes | # MiRNAs |
|---|---|---|---|---|
| 1 | Pathways in cancer | 8.22E-34 | 190 | 49 |
| 2 | PI3K-AKT signaling pathway | 1.49E-33 | 206 | 49 |
| 3 | MAPK signaling pathway | 1.43E-26 | 155 | 46 |
| 4 | Endocytosis | 1.24E-21 | 118 | 45 |
| 5 | Focal adhesion | 3.00E-21 | 127 | 47 |
| 6 | Regulation of actin cytoskeleton | 1.10E-20 | 116 | 45 |
| 7 | WNT signaling pathway | 4.65E-18 | 98 | 47 |
| 8 | Chemokine signaling pathway | 1.87E-17 | 102 | 47 |
| 9 | Hepatitis B | 3.58E-17 | 90 | 45 |
| 10 | Axon guidance | 5.81E-16 | 82 | 44 |
| 11 | Ubiquitin mediated proteolysis | 1.09E-14 | 84 | 44 |
| 12 | Insulin signaling pathway | 1.09E-14 | 85 | 49 |
| 13 | RNA transport | 1.69E-14 | 82 | 43 |
| 14 | Neurotrophin signaling pathway | 1.69E-14 | 82 | 46 |
| 15 | Measles | 1.92E-14 | 79 | 43 |

KEGG = Kyoto Encyclopedia of Genes and Genomes.

**Supplementary Table S3: Top 15 and 12 miRNAs observed up- and down-regulated, respectively, in the TNBC subtype in comparison to the non-TNBC subtype of the NHW group of patients**

| MiRNAs Up-regulated | Log2 FC | *P*-value | FDR | MiRNAs down-regulated | Log2 FC | *P*-value | FDR |
|---|---|---|---|---|---|---|---|
| hsa-miR-522-3p | 8.41 | 1.46E-18 | 0 | hsa-miR-1253 | –4.45 | 2.99E-07 | 2.78E-06 |
| hsa-miR-451a | 7.12 | 8.63E-12 | 8.63E-10 | hsa-miR-1469 | –3.26 | 1.84E-07 | 1.82E-06 |
| hsa-miR-150-5p | 6.68 | 2.61E-09 | 6.14E-08 | hsa-miR-548aa | –2.74 | 1.18E-08 | 2.01E-07 |
| hsa-miR-126-3p | 6.53 | 3.38E-10 | 1.18E-08 | hsa-miR-520d-5p+ miR-518a-5p+miR-527 | –2.52 | 1.79E-07 | 1.81E-06 |
| hsa-miR-223-3p | 6.41 | 3.29E-10 | 1.20E-08 | hsa-miR-1234 | –2.25 | 5.18E-06 | 2.82E-05 |
| hsa-miR-374a-5p | 6.01 | 1.00E-10 | 6.17E-09 | hsa-miR-877-5p | –1.89 | 2.01E-05 | 9.20E-05 |
| hsa-miR-125a-5p | 5.98 | 8.25E-12 | 9.42E-10 | hsa-miR-376a-3p | –1.81 | 1.33E-06 | 8.87E-06 |
| hsa-miR-651 | 5.95 | 1.79E-13 | 4.78E-11 | hsa-miR-874 | –1.50 | 3.11E-04 | 9.73E-04 |
| hsa-miR-182-5p | 5.75 | 4.61E-13 | 9.22E-11 | hsa-miR-1224-5p | –1.50 | 8.42E-04 | 0.002 |
| hsa-miR-762 | 5.70 | 2.31E-12 | 3.07E-10 | hsa-miR-940 | –1.49 | 0.001 | 0.003 |
| hsa-miR-34a-5p | 5.60 | 1.78E-10 | 7.50E-09 | | | | |
| hsa-miR-185-5p | 5.55 | 2.84E-14 | 1.14E-11 | | | | |
| hsa-miR-181a-5p | 5.19 | 8.43E-09 | 1.57E-07 | | | | |
| hsa-miR-18a-5p | 5.16 | 5.85E-11 | 4.68E-09 | | | | |
| hsa-miR-191-5p | 5.13 | 2.56E-09 | 6.20E-08 | | | | |

FC = fold change, FDR = false discovery rate.

**Supplementary Table S4: Top 15 KEGG pathways (based on P value) mostly affected by the differentially expressed miRNAs in the TNBC and non-TNBC subtypes of the NHW group of patients (DIANA miRPath v.2.0)**

| # | KEGG pathway | *P*-value | # Genes | # MiRNAs |
|---|---|---|---|---|
| 1 | Pathways in cancer | 5.02E-43 | 191 | 43 |
| 2 | PI3K-AKT signaling pathway | 2.95E-38 | 181 | 44 |
| 3 | MAPK signaling pathway | 2.27E-34 | 159 | 45 |
| 4 | HTLV-I infection | 5.00E-30 | 137 | 45 |
| 5 | Endocytosis | 1.37E-27 | 119 | 39 |
| 6 | Regulation of actin cytoskeleton | 1.44E-27 | 124 | 39 |
| 7 | Epstein-Barr virus infection | 1.92E-24 | 109 | 37 |
| 8 | Focal adhesion | 1.46E-23 | 110 | 41 |
| 9 | Transcriptional misregulation in cancer | 2.08E-21 | 102 | 43 |
| 10 | WNT signaling pathway | 2.38E-21 | 93 | 41 |
| 11 | Chemokine signaling pathway | 4.49E-21 | 97 | 40 |
| 12 | Axon guidance | 1.40E-20 | 82 | 38 |
| 13 | Hepatitis B | 2.68E-20 | 80 | 38 |
| 14 | Neurotrophin signaling pathway | 1.27E-18 | 83 | 41 |
| 15 | Ubiquitin mediated proteolysis | 3.19E-18 | 82 | 40 |

KEGG = Kyoto Encyclopedia of Genes and Genomes.

**Supplementary Table S5: Area Under the Curve (AUC) values of the individual 26 miRNAs differentially expressed between the AA and NHW-TNBC group of patients**

| MiRNA | AUC (95% CI) |
|---|---|
| hsa-miR-1225-3p | 0.89 (0.81–0.98) |
| hsa-miR-216a-5p | 0.88 (0.78–0.97) |
| hsa-miR-532-3p | 0.87 (0.77–0.97) |
| hsa-miR-580-3p | 0.87 (0.77–0.97) |
| hsa-miR-599 | 0.86 (0.75–0.96) |
| hsa-miR-769-5p | 0.86 (0.75–0.98) |
| hsa-miR-18a-5p | 0.84 (0.73–0.95) |
| hsa-miR-28-5p | 0.83 (0.70–0.95) |
| hsa-miR-182-5p | 0.83 (0.71–0.94) |
| hsa-miR-183-5p | 0.83 (0.72–0.94) |
| hsa-miR-205-5p | 0.82 (0.69–0.94) |
| hsa-miR-4284 | 0.83 (0.72–0.95) |
| hsa-miR-4458 | 0.83 (0.71–0.95) |
| hsa-miR-614 | 0.82 (0.70–0.94) |
| hsa-miR-940 | 0.80 (0.67–0.92) |
| hsa-miR-1263 | 0.80 (0.68–0.93) |
| hsa-miR-4431 | 0.80 (0.68–0.92) |
| hsa-miR-15b-5p | 0.79 (0.66–0.91) |
| hsa-miR-17-5p | 0.79 (0.67–0.92) |
| hsa-miR-93-5p | 0.79 (0.66–0.91) |
| hsa-miR-548ad-3p | 0.79 (0.66–0.92) |
| hsa-miR-661 | 0.79 (0.65–0.92) |
| hsa-miR-23a-3p | 0.78 (0.64–0.91) |
| hsa-miR-200c-3p | 0.78 (0.64–0.91) |
| hsa-miR-3934-5p | 0.78 (0.65–0.91) |
| hsa-miR-150-5p | 0.75 (0.61–0.89) |
| **Score 1** (combined panel) | **0.88 (0.78–0.99)** |
